# Robotic-assisted complete mesocolic excision, central vascular ligation and para-aortic lymph node dissection in multifocal carcinoid: A case report and technical description

**DOI:** 10.1016/j.ijscr.2020.02.018

**Published:** 2020-02-11

**Authors:** R. Young, A. Rajkomar, P. Smart, S. Warrier

**Affiliations:** a Department of Surgery, Melbourne Health, Melbourne, Victoria, Australia; b Gastrointestinal Clinical Institute, Epworth Healthcare, Victoria, Australia; c Department of Surgery, Austin Health, Victoria, Australia; d Department of Cancer Surgery, Peter MacCallum Cancer Centre, Victoria, Australia; e Department of Surgery, Alfred Health, Victoria, Australia

**Keywords:** Robotic surgical procedures, Lymph node excision, Case report

## Abstract

**Introduction:**

Neuroendocrine tumours are the most common type of primary small bowel neoplasm. Consensus guidelines recommend a multimodal approach to treatment of such tumours, with aggressive surgical resection remaining the mainstay of management. There is evidence that complete mesocolic excision (CME) of lymph nodes is associated with superior oncological outcomes including longer disease-free survival in patients with colorectal cancer than standard lymph node dissection and there is increasing evidence to suggest that the robotic approach may be superior to laparoscopic or open CME. This report discusses a robotic-assisted approach to CME with central vessel ligation (CVL) and para-aortic lymph node dissection in a case of multifocal neuroendocrine tumour of the small bowel.

**Presentation of case and technical approach:**

This report details the case of a 73-year-old male with multifocal small bowel neuroendocrine tumour. He underwent a robotic-assisted right hemicolectomy, small bowel resection, CME, CVL and para-aortic lymph node dissection. The approach described involved undertaking CME, CVL and bowel resection with a standard right hemicolectomy robotic set-up before re-docking the robot to perform the retroperitoneal para-aortic lymph node dissection.

**Discussion:**

This case highlights the management of multifocal small bowel neuroendocrine tumour using a robotic approach for surgical resection and lymph node clearance.

**Conclusion:**

The robotic approach provides a safe and effective technique for undertaking surgical resection of small bowel neuroendocrine tumour as well as complete mesocolic excision of lymph nodes. With a change in port positions, a robotic approach can be utilised for CME/CVL as well as retroperitoneal node dissection.

## Highlights

• Neuroendocrine tumours are the most common type of small bowel neoplasm.
• Robotic technique may be superior to open technique for lymph node dissection.
• Robotic-assisted complete mesocolic excision is a safe and effective technique.

## 1 Introduction

Neuroendocrine tumours (NET) are the most common primary small bowel tumours accounting for around 30 % of small bowel neoplasms [1,2]. NETs are often slow growing, typically asymptomatic and often difficult to identify on conventional diagnostic imaging due to their location. As such, they are often diagnosed late, once they have progressed to an advanced stage and are causing symptoms such as obstruction, pain, bleeding or carcinoid crisis [3].

Midgut neuroendocrine tumours (tumours located in the jejunum or ileum) and particularly multifocal midgut NETs are the most common type of NET to develop distant metastases [3]. Gangi et al. reported presence of microscopic or macroscopic mesenteric lymph node metastasis in 74.4 % of patients with small bowel NETS with metastatic disease being most common in multifocal tumours [2]. Despite their frequently advanced stage, survival time for metastatic NET remains long with a median survival of 56 months [3].

Consensus recommendations suggest a multimodal approach to managing small bowel NETs with aggressive surgical management as the mainstay of treatment [3]. Lymph node clearance is recommended as part of the standard surgical approach for small bowel NET as 46–98 % of patients with small bowel NET are found to have lymph node involvement following operative dissection [3]. There is currently no standard practice guideline to suggest the extent of lymph node dissection required in small bowel NET, but complete mesocolic excision (CME) is increasingly being undertaken as there is evidence to suggest that it is associated with superior oncological outcomes including longer disease-free survival in patients with colorectal cancers than non-CME [4,5].

Practitioners are increasingly utilising a robotic technique for complete mesocolic excision (CME) and central vascular ligation (CVL) and there is mounting evidence that a minimally-invasive approach provides superior outcomes when compared to open technique for CME [[6], [7], [8], [9]]. With the benefit of factors such as 3D vision, arm stability and instrument range of motion, robotic-assisted surgery may provide superior outcomes compared to a laparoscopic approach, although the evidence behind a robotic-assisted approach to CME remains limited. This case demonstrates a technical approach to undertaking bowel resection, CME and CVL and retroperitoneal lymph node dissection which does not require patient repositioning intra-operatively.

This work has been reported in line with the SCARE criteria [10].

## 2 Case report

**Fig. 1:**
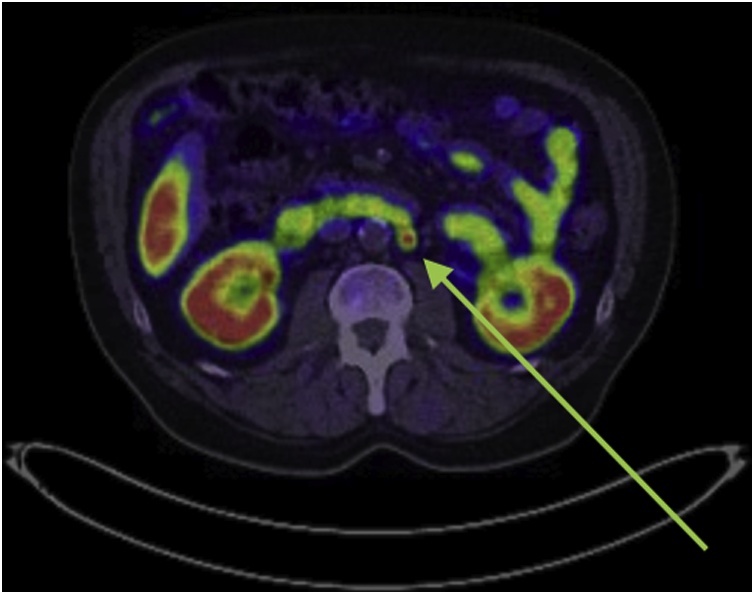
Pre-operative PET DOTATE scan of involved retroperitoneal lymph node.

**Fig. 2:**
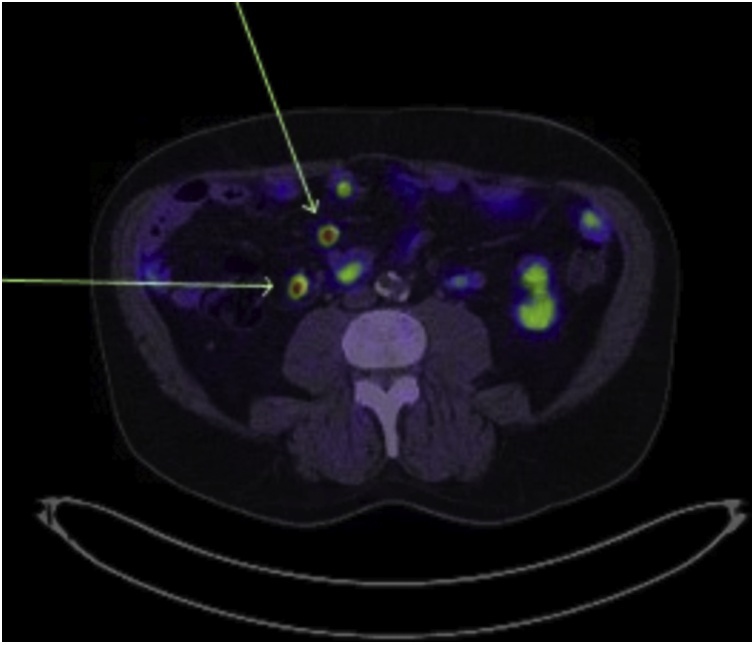
Pre-operative PET DOTATE scan of involved ileal mesenteric lymph nodes.

**Fig. 3:**
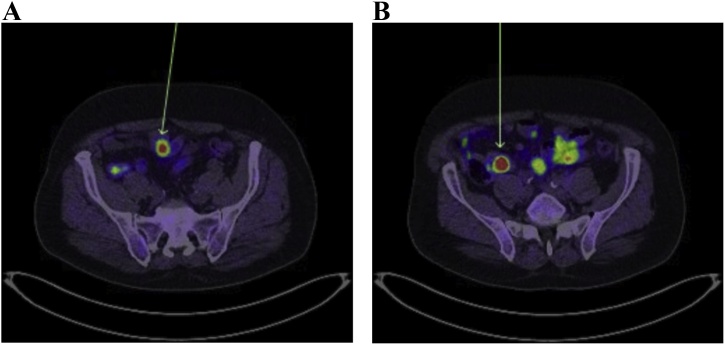
Pre-operative PET DOTATE scan demonstrating a) jejunal NET b) most advanced ileal NET.

A 73-year-old male, with a past medical history significant for type two diabetes, ischaemic heart disease and atrial fibrillation presented with iron-deficiency anaemia. He was obese with a BMI of 32.5. Upper GI endoscopy and colonoscopy were unrevealing. He subsequently underwent a capsule endoscopy which revealed several ulcerated submucosal lesions in the proximal and distal ileum. Biopsy of the most distal lesion was undertaken via retrograde double-balloon enteroscopy (DBE) and this revealed the diagnosis of a well-differentiated neuroendocrine tumour. The two more proximal lesions seen on capsule endoscopy were not visualised during DBE. A Positron Emission Tomography (PET) DOTA-TATE scan was undertaken which revealed the presence of avid lymph nodes in the ileal mesentery as well a single, 9 mm avid node in the retroperitoneum adjacent to the aorta (Fig. 1, Fig. 2, Fig. 3). Urinary 5-HIAA level was normal and serum chromogranin-A level was 235 units/L in the context of proton-pump inhibitor use.

The operative approach taken was a robotic right hemicolectomy with complete mesocolic excision, small bowel resection, central vascular ligation and retroperitoneal para-aortic lymph node dissection using the Da Vinci Xi® Surgical System (Intuitive Surgical Inc., Sunnyvale, CA, USA). Surgery was undertaken by two, experienced colorectal surgeons in a private hospital setting.

## 3 Technical approach

**Fig. 4:**
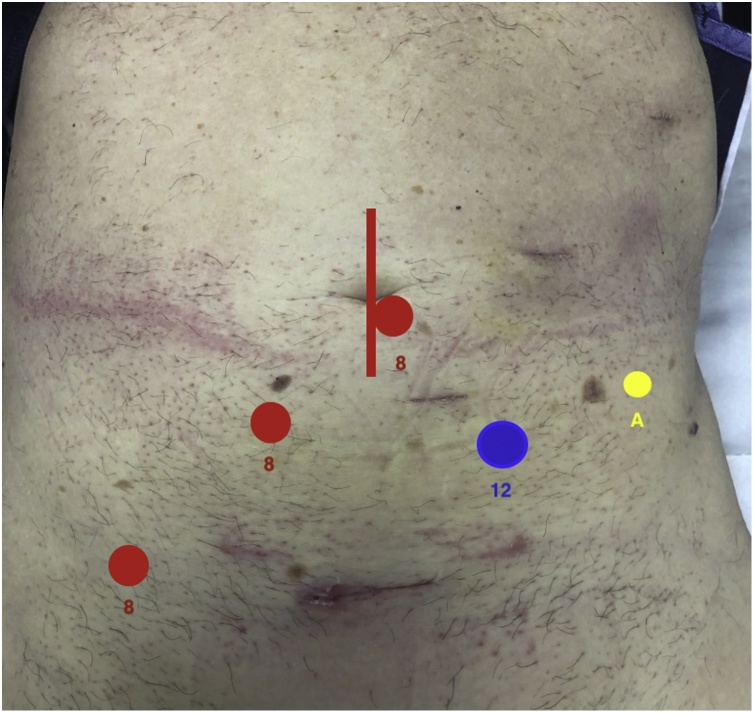
Port placement and set-up for robotic CME, CVL and bowel resection. Working arms in right lower abdomen and suprapubic region, camera in midzone and assistant port in left lower quadrant (left to right in image).

The patient was positioned supine on the operating table with a standard four port robotic Xi technique adopted (see Fig. 4). A line between the patient’s left midclavicular line and the right ischium was used as a guide for the four ports. An additional assistant port was placed in the left iliac fossa. For the CME dissection, arm 3 was used as the camera port and targeting was aimed at the hepatic flexure. Arms 1 and 2 were used as left-handed retracting instruments while arm 4 was used as the main dissector (advanced bipolar energy or monopolar cautery with scissors). A superior mesenteric vein (SMV) first technique was adopted for the CME and CVL dissection whereby all lymphatic tissue overlying the SMV was removed. The ileocolic artery was seen running anterior to the SMV in this case and therefore ligated proximally to facilitate more cranial dissection. The dissection was continued to the right colic vein (RCV) and up to the gastrocolic trunk of Henle. Given the burden of disease on imaging was ileal in nature, a decision was made not to remove the right branch of the middle colic vessels and perform a more targeted CVL. Upon completion of the vascular dissection, the rest of the anatomical dissection was completed. This was performed with a medial to lateral approach that used embryological planes and respected the underlying retroperitoneal structures. A standard robotic ileocolic intracorporeal anastomosis was performed with a 60 mm blue load Sureform®™ stapler and 3-0 V-Loc™ to close the common enterotomy. Upon completion of this stage, attention turned to the retroperitoneal dissection.

**Fig. 5:**
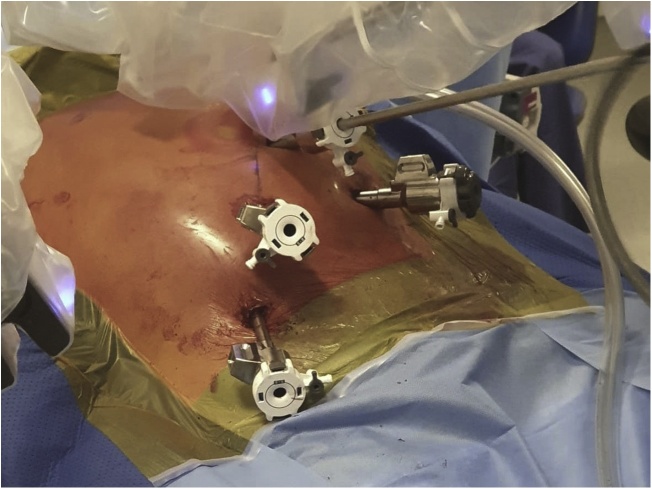
Port placement and set-up for robotic para-aortic lymph node dissection.

**Fig. 6:**
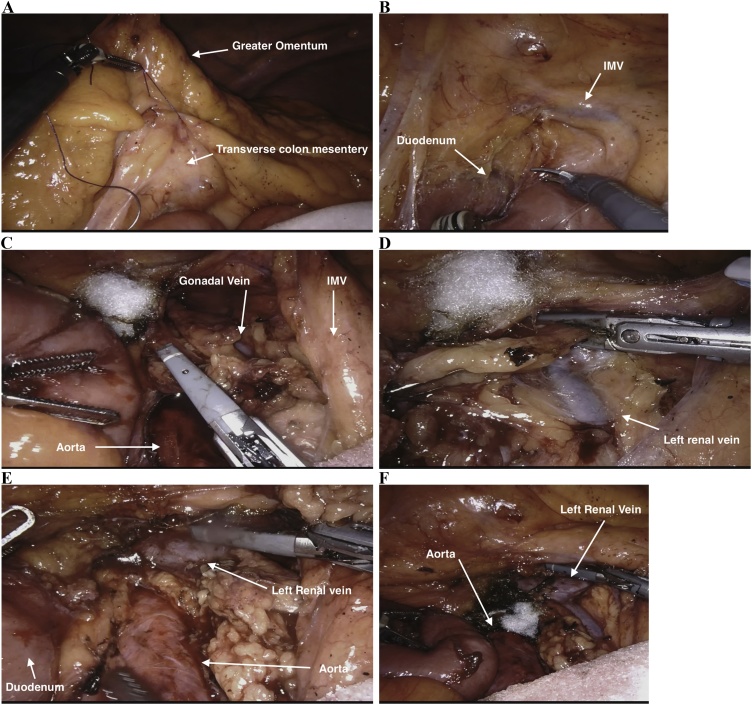
Intra-operative photographs demonstrating sequence of robotic RPLND a) suture transverse colon mesentery to anterior abdominal wall b) medialisation of duodenal-jejunal flexure c) exposure of aorta and left gonadal vein d) dissection along left gonadal vein to left renal vein e) exposure of left renal vein f) completed dissection.

In order to facilitate the robotic retroperitoneal lymph node dissection, arms 3 and 4 were de-docked. The laparoscope was transferred to arm 2 and the assistant port converted to arm 3. Arm 4 was used as the assistant port for this stage of the dissection (see Fig. 5). The robot was re-targeted towards the falciform ligament. The appendices epiploacae of the transverse colon were sutured to the abdominal wall to lift the transverse mesocolon vertically and provide retraction. A further fixation suture was placed to retract the distal transverse mesocolon thereby elevating the IMV. A reverse kocherisation of the duodenum off the aorta was followed by a complete para-aortic lymphadenectomy (the medial boundary being the inferior vena cava (IVC), the lateral boundary the left gonadal vein, the superior boundary the left renal vein and the distal boundary being the inferior mesenteric artery (IMA). Once the lymphadenectomy was complete the robot was undocked and a 3 cm periumbilical midline incision was made. The ileocolic specimen was retrieved. The small bowel was run and the jejunal carcinoid specimens were located by palpating the small bowel, as these lesions were not visible macroscopically. A small bowel resection and hand sewn small bowel anastomosis performed. The surgery was completed without complication and the patient was discharged home on day three post-operatively (Fig. 6).

Histopathology from the operative resection demonstrated T1N1M0 Grade 2 multifocal neuroendocrine tumour in the terminal ileum and jejunum with a total of 8 deposits of grade small bowel NET. Six of the twenty-two excised lymph nodes demonstrated metastatic tumour deposits.

## 4 Discussion

This case highlights the use of a robotic approach for the surgical management of multifocal small bowel neuroendocrine tumour.

Robotic surgery offers advantages compared with traditional laparoscopic techniques in terms of 3D visualisation and improved instrument dexterity. Increasingly, robotic-assisted techniques are being employed for surgical resection of gastrointestinal tract tumours and the associated lymph node dissection [11]. While the evidence to support a robotic-assisted approach is still limited, there is mounting evidence to suggest that this technique is associated with superior oncological and post-operative outcomes than open surgery. Practitioners have been slow to adopt a robotic-assisted technique as standard practice in the surgical management of colorectal cancer for several reasons. Robotic surgery is associated with increased cost and longer operative times than open or laparoscopic techniques, especially in the initial learning curve. Access to robotic technology is as yet, not available in all centres and there are still many surgeons without experience using a robotic technique. Despite these challenges, there is increasing evidence to suggest that there is greater lymph node yield, superior oncological resection and longer disease-free survival when using a robotic-assisted approach to surgical resection in patients with colorectal cancer [5,8,12]. As such, this technique should be considered and offered, where possible to patients with colorectal cancer requiring surgical resection.

There have been concerns about utilising a robotic-assisted approach for undertaking retroperitoneal lymph node dissection in the literature with some authors describing the need for patient repositioning and robotic re-docking intra-operatively to perform a thorough, bilateral retroperitoneal node dissection. There is evidence however in the literature to suggest that with careful port positioning, a complete retroperitoneal node dissection can be undertaken without changing patient position [13]. This case report adds to the evidence by demonstrating how, with a small change in port position, bowel resection, CME and retroperitoneal node dissection can all be undertaken without needing to significantly reposition intra-operatively.

This report describes use of a robotic technique in a case of multifocal carcinoid. It adds to the growing body of evidence to support this approach for surgical resection of bowel cancers [3].

## 5 Conclusion

Small bowel NETs are the most common primary small bowel neoplasm. Management of these tumours is best undertaken using a multimodal approach with aggressive surgical resection being the mainstay of treatment. The robotic approach provides a safe and effective means of undertaking both bowel resection and extensive lymph node dissection in cases of multifocal small bowel NETs.

## Sources of funding

This study was supported by Epworth Research Institute Major Research Grant No. 11.952.000.80982.

## Ethical approval

This study has been exempt from ethical approval at our institution, however, permission has been sought from the patient in question to present and publish this case report.

## Consent

Consent has been obtained (written and signed) from the patient in question to complete this case report.

## Author’s contribution

Dr Rebekah Young: Conceptualisation, writing of original draft.

Mr Amrish Rajkomar: manuscript editing.

Mr Phil Smart: Data curation, conceptualisation, funding acquisition, review and editing of manuscript.

Mr Satish Warrier: Data curation, conceptualisation, funding acquisition, review and editing of manuscript.

## Registration of research studies

Not applicable – case report only.

## Guarantor

Mr Phillip Smart.

## Disclosure statement

This paper is not based on a communication to a society or meeting. The authors have no conflict of interest or financial ties to disclose.

## Provenance and peer review

Not commissioned, externally peer-reviewed.

## Declaration of Competing Interest

Nil to declare.
